# Residual Mechanical and Structural Properties of Non-Calcined Hwangto Concrete After Exposure to High Temperatures

**DOI:** 10.3390/ma19040724

**Published:** 2026-02-13

**Authors:** Taehyung Kim, Wonchang Kim, Hajun Im, Taegyu Lee

**Affiliations:** Department of Fire and Disaster Prevention, Semyung University, Jecheon 27136, Republic of Korea; kth1514@gmail.com (T.K.); firesafety9505@gmail.com (W.K.); haha1578@naver.com (H.I.)

**Keywords:** mineral admixture, high temperature, Hwangto, residual mechanical properties, dissipated energy

## Abstract

This study evaluated the residual mechanical properties of concrete in which Ordinary Portland Cement (OPC) was partially replaced with non-calcined Hwangto (NHT). Specimens were prepared with two water-to-binder (W/B) ratios (0.41 and 0.33) and three NHT replacement levels (0%, 15%, and 30%). The specimens were exposed to elevated temperatures of 20, 100, 200, 300, 500, and 700 °C at a heating rate of 1 °C/min. The results indicated that while the initial compressive strength at room temperature decreased with increasing NHT content, the residual mechanical performance at high temperatures significantly improved. Notably, temporary strength recovery was observed in the 200–300 °C range due to the internal autoclaving effect. At 700 °C, the NHTC (non-calcined Hwangto concrete)-30 series exhibited the highest thermal stability, retaining 28.2% of its initial compressive strength, whereas the Plain (OPC Concrete) and NHTC-15 series retained only 23.6% and 22.4%, respectively. Regarding energy absorption, the dissipated energy varied with the W/B ratio. In the W/B 41 series, the NHTC-30 specimen demonstrated superior ductility and energy dissipation capacity at 700 °C, outperforming the Plain specimen. This enhanced post-peak performance is attributed to the thermal activation of kaolinite into metakaolin, which preserves microstructural integrity by mitigating the severe degradation of hydration products and inhibiting crack propagation. These findings suggest that incorporating NHT effectively enhances the fire resistance and residual structural integrity of concrete, particularly in normal-strength matrices.

## 1. Introduction

Concrete is the most widely used construction material globally. However, its conventional reliance on cement poses significant environmental challenges. With the growing urgency to mitigate greenhouse gas emissions, which are key drivers of extreme weather events, international organizations have established ambitious targets such as “Net Zero by 2050” [1].

Cement production is carbon-intensive, generating approximately 0.58 tons of CO_2_ per ton of cement. A substantial portion (60–65%) of these emissions originates from the calcination of limestone to produce CaO, with the remainder largely attributed to the thermal energy required to maintain kiln temperature at 1450 °C [2]. To address this, the immediate global strategy involves reducing the clinker factor by incorporating mineral admixtures. These admixtures, which include industrial by-products and pozzolans like kaolin and clay, replace a portion of the cement in concrete. Beyond reducing carbon emissions, they can enhance mechanical properties and durability by promoting pozzolanic reactions and refining pore structures to inhibit chloride penetration. Furthermore, their economic feasibility makes them attractive candidates for high-performance, cost-effective concrete [3,4,5,6,7,8,9].

In densely populated regions like South Korea, the prevalence of high-rise residential buildings has heightened fire safety concerns. To improve energy efficiency, Exterior Insulation Finishing Systems (EIFSs) using combustible materials (e.g., EPS, XPS) have been widely adopted. However, these materials can accelerate fire spread, as evidenced by numerous recent incidents [10]. Fire exposure degrades concrete by altering its mechanical properties and energy dissipation capacity, complicating post-fire structural assessment. Therefore, evaluating the residual mechanical properties of concrete after high-temperature exposure is critical for ensuring structural safety and preventing disasters [11].

Research on material failure mechanisms and energy dissipation is fundamental to disaster prevention, extending beyond construction to fields such as rock mechanics. For instance, Lin et al. investigated the failure characteristics of coal under dynamic loading to mitigate geological disasters [12]. They visualized the failure process in four distinct stages: the proliferation of micro-cracks, the formation of macro-cracks, crack propagation, and final collapse [12].

Analogous to these failure mechanisms, high temperatures typically induce macro- and micro-cracking in concrete due to the differential thermal expansion between cement paste and aggregates, leading to mechanical deterioration [13,14,15,16,17]. The degree of damage depends on mix parameters such as the water-to-binder (W/B) ratio and the type of binder or aggregate used. Notably, certain mineral admixtures with lower thermal expansion coefficients than Ordinary Portland Cement (OPC) are being investigated for their potential to improve fire resistance. For instance, Poon et al. found that concrete with 5% metakaolin outperformed mixes with fly ash or silica fume, although higher replacement levels (10–20%) reduced residual strength [15]. Similarly, Saridemir reported that concrete containing ground pumice and metakaolin maintained residual strengths comparable to or higher than those of OPC [16]. Most existing studies on kaolin-based materials have focused on calcined kaolin, which has demonstrated excellent residual mechanical properties [17,18,19,20,21,22,23].

Hwangto, a kaolinitic mineral covering approximately 10% of the Earth’s surface, is another potential silica-rich pozzolan. However, its application in concrete has been limited by low strength development, attributed to high water absorption and the agglomeration of unreacted particles that weaken interfacial bonds [24]. Despite this, Hwangto exhibits high heat resistance, as it chemically decomposes at temperatures higher than those of cement hydrates [25,26].

While previous research has focused heavily on calcined kaolin or clay, there is a pressing need to investigate non-calcined materials to eliminate the energy consumption and emissions associated with the sintering process. Although non-calcined materials typically exhibit lower initial strength, it is hypothesized that high-temperature exposure may induce thermal effects that enhance their mechanical performance, like the behavior of calcined kaolin. Therefore, this study analyzes the high-temperature mechanical properties of concrete incorporating non-calcined Hwangto (NHT) as a mineral admixture.

## 2. Experimental Procedure

### 2.1. Experimental Program

Table 1 summarizes the experimental program. Cylindrical concrete specimens (Φ100 × 200 mm) were prepared by replacing Ordinary Portland Cement (OPC) with NHT. The experimental variables included three NHT replacement ratios (0%, 15%, and 30%), two water-to-binder (W/B) ratios (0.41 and 0.33), and six target temperatures, including ambient temperature. The heating rate was set at 1 °C/min, followed by a 60 min holding period at the target temperature. After exposure to elevated temperatures, the residual mechanical properties of each specimen, including unit weight, compressive strength, stress–strain relationship, and dissipated energy, were evaluated. To ensure statistical reliability, three replicate specimens were tested for each mix proportion and target temperature. The results presented in this study represent the average values of these three measurements.

### 2.2. Materials

Table 2 and Table 3 present the physical and chemical properties of the materials used in this experiment, respectively. Ordinary Portland Cement (Yeongwol, Republic of Korea) with a density of 3.15 g/cm^3^ and fineness of 3200 cm^2^/g was used as the binder. Non-calcined Hwangto (Yeongam, Republic of Korea), incorporated as a mineral admixture to partially replace cement, had a density of 2.50 g/cm^3^ and a fineness of 3300 cm^2^/g. Crushed granite with a maximum aggregate size of 20 mm was used as the coarse aggregate; it had a density of 2.68 g/cm^3^, a fineness modulus of 7.03, and a water absorption rate of 0.68%. River sand was used as the fine aggregate, with a density of 2.54 g/cm^3^, a fineness modulus of 2.54, and a water absorption rate of 1.60%. A polycarboxylate-based superplasticizer was added to ensure consistent workability.

### 2.3. Mix Proportions

Table 4 summarizes the mix proportions for the six concrete mixtures. To investigate the effect of NHT on mechanical properties, two water-to-binder (W/B) ratios (0.41 and 0.33) were selected, with corresponding sand-to-aggregate (S/a) ratios fixed at 46% and 43%, respectively. NHT was used to replace cement at three levels: 0%, 15%, and 30% by weight. The replacement ratios for NHT were determined based on previous studies regarding kaolin-based mineral admixtures. For instance, Mo et al. reported that incorporating 10–15% calcined kaolin clay (CKC) into ultra-high-performance concrete (UHPC) yields optimal mechanical strength, whereas higher dosages (e.g., 20%) can lead to performance degradation due to the adverse effects of excessive mineral admixture incorporation [8]. Conversely, regarding high-volume replacement, Du et al. demonstrated that a 30% substitution of cement with calcined clay and limestone in high-performance concrete (HPC) results in mechanical properties comparable to Ordinary Portland Cement (OPC) after 7 days, validating 30% as a feasible upper threshold for sustainable binder design [9]. Guided by these findings, this study selected 15% to represent the optimal dosage for mechanical enhancement and 30% to evaluate the viability of high-volume utilization for maximizing carbon reduction. Furthermore, adopting these established replacement ratios facilitates a direct comparison with the existing literature on calcined clays, allowing for a critical assessment of the “in situ thermal activation” efficiency of NHT under fire conditions. The resulting mixtures were designated as Plain, NHTC-15, and NHTC-30. Cylindrical specimens (Φ100 × 200 mm) were cast for all mixtures. After 24 h of casting, the specimens were demolded and water-cured for 28 days. Subsequently, they were air-cured in a controlled environment (20 ± 2 °C, 60 ± 5% RH) until the testing age of 91 days. The testing age of 91 days was selected to evaluate the long-term pozzolanic contribution of NHT. Unlike Ordinary Portland Cement (OPC), which achieves most of its hydration within 28 days, mineral admixtures typically exhibit slower reaction kinetics. Extending the curing period to 91 days allows for the sufficient development of secondary C-S-H gels through the reaction between NHT and calcium hydroxide, ensuring a stable and mature microstructure prior to high-temperature exposure.

### 2.4. Heating and Testing Methods

**Figure 1:**
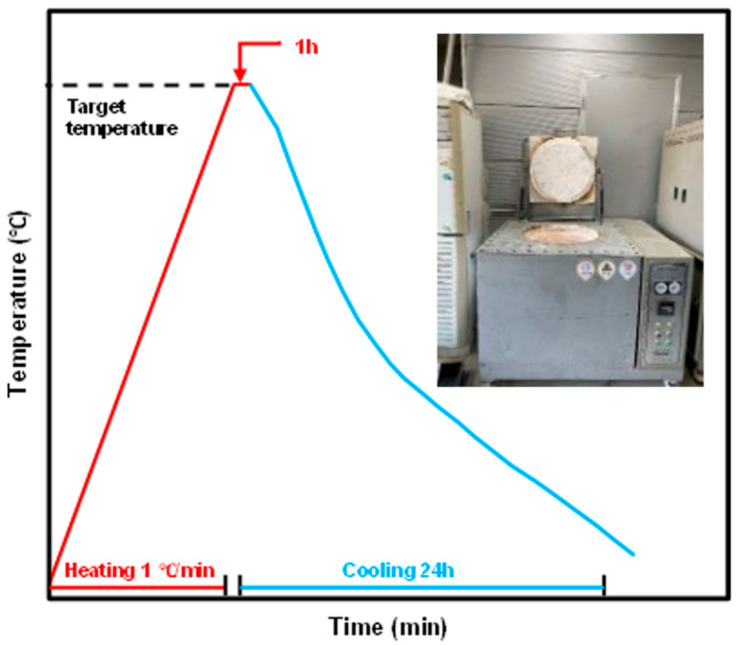
The heat rate and furnace used in the test [27].

Figure 1 illustrates the electric furnace and heating method used to evaluate high-temperature mechanical properties. The target temperatures were set at six levels: 20 °C (ambient), 100 °C, 200 °C, 300 °C, 500 °C, and 700 °C. A heating rate of 1 °C/min was maintained to ensure uniform temperature distribution within the specimens, followed by a 60 min holding period at the target temperature.

The measured parameters included unit weight, compressive strength, stress–strain relationship, and dissipated energy. All mechanical tests were conducted at a concrete age of 91 days, with compressive strength measured in accordance with ASTM C39/39M.

To evaluate the energy absorption capacity of specimens exposed to high temperatures, the dissipated energy was calculated based on previous studies [26,28]. In this study, dissipated energy is defined as the area under the stress–strain curve up to the peak stress (ultimate strain) where macroscopic cracking initiates. Although this method is typically applied to fiber-reinforced or reinforced concrete, it was adapted here to compare the residual energy absorption capacity of unreinforced concrete containing different NHT ratios under identical thermal conditions. Since unreinforced concrete is inherently brittle, the energy calculation was strictly limited to the pre-peak region of the stress–strain curve.

## 3. Results

### 3.1. TGA and Unit Weight

**Figure 2:**
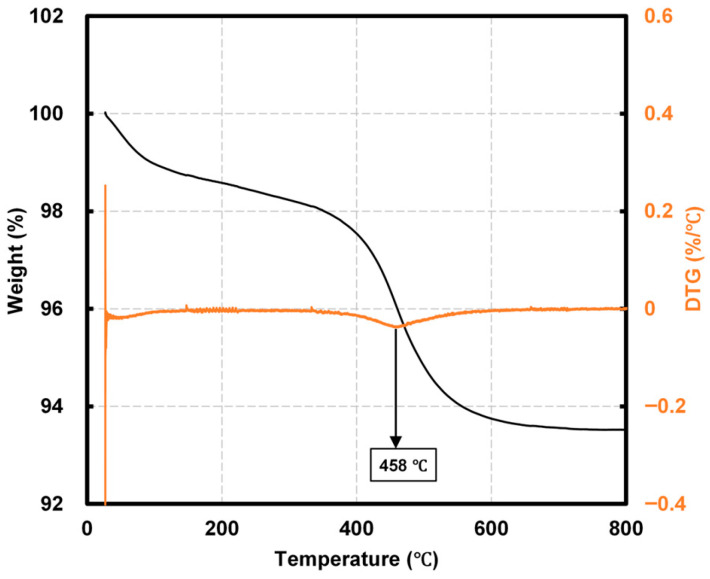
TGA results for NHT.

Figure 2 presents the Thermogravimetric Analysis (TGA) results for NHT. Up to 200 °C, a weight loss of approximately 1.41% was observed, which is attributed to the evaporation of absorbed water from the surface of the Hwangto particles. Between 200 °C and 400 °C, a further weight loss of about 2.45% occurred, accompanied by slight fluctuations in the Derivative Thermogravimetry (DTG) curve. A distinct DTG peak appeared at approximately 458 °C, followed by a rapid weight reduction up to 600 °C, resulting in a total weight loss of 6.47%. Azizi et al. reported that rapid weight loss in the 400–600 °C range is closely associated with the dehydroxylation of kaolinite, a process critical for metakaolin formation and pozzolanic activity [29]. According to Kim et al., high-purity (95%) kaolinite typically exhibits a DTG peak between 500 and 600 °C and a weight loss of approximately 12% in the 600–800 °C range—significantly higher temperatures and mass loss than those observed in this study [30]. Consequently, the relatively lower weight loss of NHT suggests that it consists of lower-purity kaolinite containing a substantial amount of heat-resistant impurities.

**Figure 3:**
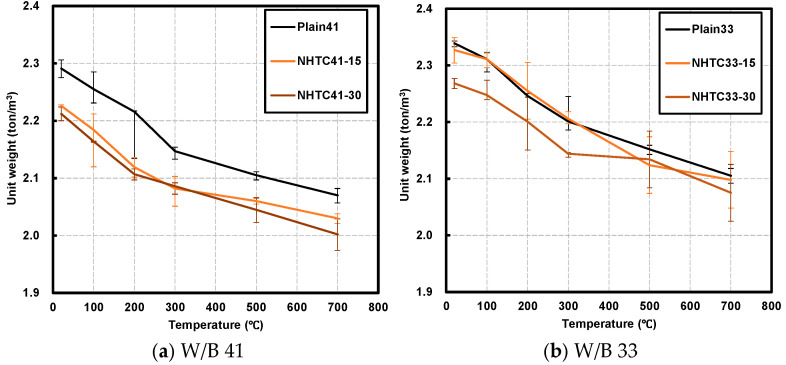
Unit weight after exposure to target temperature.

Figure 3 illustrates the variations in the unit weight of the heated concrete specimens. Due to the lower specific gravity of NHT (2.50) compared to OPC (3.15), the initial unit weight decreased as the NHT replacement level increased.

In the W/B 41 series, the unit weight reductions for the Plain, NHTC-15, and NHTC-30 specimens were 1.6%, 1.9%, and 2.2% at 100 °C; 3.3%, 4.8%, and 4.7% at 200 °C; 6.3%, 6.5%, and 5.7% at 300 °C; 8.1%, 7.5%, and 7.5% at 500 °C; and 9.6%, 8.8%, and 9.5% at 700 °C, respectively. Similarly, the W/B 33 series exhibited reductions of 1.2–0.9% at 100 °C, 4.0–3.0% at 200 °C, 5.9–5.5% at 300 °C, 8.0–5.9% at 500 °C, and 10.0–8.5% at 700 °C.

A significant mass loss was observed around 200 °C, which is primarily attributed to the evaporation of free and physically bound water within the concrete matrix. Furthermore, a pronounced mass loss occurred at approximately 500 °C. For the OPC control, this is mainly due to the decomposition of hydration products such as calcium hydroxide (portlandite) [20]. However, in the NHT-incorporated specimens, this reduction is likely intensified by the dehydroxylation of kaolinite, consistent with the DTG peak at 458 °C observed in Figure 2. The average unit weight reductions for the W/B 41 series at the target temperatures (100–700 °C) were 1.9%, 4.8%, 6.2%, 7.7%, and 9.3%, whereas the W/B 33 series showed lower reductions of 0.9%, 3.4%, 5.5%, 7.5%, and 9.5%. The W/B 41 series exhibited higher reduction rates at all temperatures except 700 °C. This is likely due to its more porous microstructure compared to the W/B 33 series, which facilitated the escape of internal moisture [31].

### 3.2. Residual Compressive Strength

**Figure 4:**
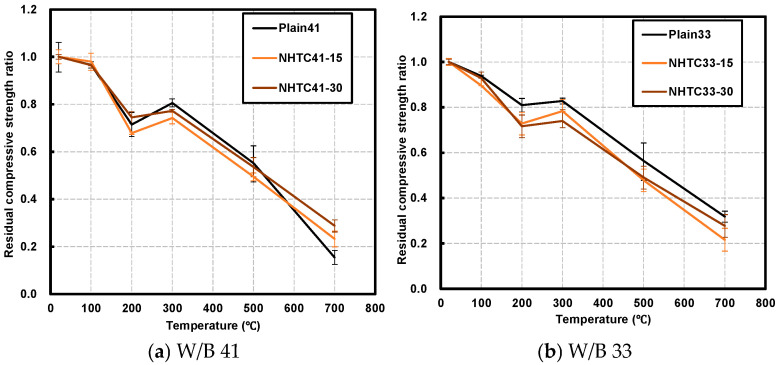
Residual compressive strength after exposure to target temperature.

Figure 4 presents the variation in the compressive strength of the specimens with respect to the target temperature. Generally, compressive strength decreased as the temperature increased relative to ambient temperature (20 °C). However, a temporary increase in compressive strength was observed in the temperature range of 200–300 °C.

Specifically, in the W/B 41 series, the NHTC-15 and NHTC-30 specimens exhibited strength increases of 6.7% and 2.0%, respectively. This trend was more pronounced in the W/B 33 series, where the NHTC-15 and NHTC-30 specimens showed increases of 7.2% and 4.6%, respectively. Consistent with previous findings by Xu [32], the strength gain was greater at the lower W/B ratio. This phenomenon is attributed to the internal autoclaving effect, where high-temperature and high-pressure steam within the concrete promotes the hydration of unhydrated cement grains or the dry hardening of the cement paste [16,23,33,34].

Beyond 400 °C, strength degradation became significant due to the dehydration of calcium hydroxide (portlandite) and the decomposition of C–S–H gel. The rate of C–S–H decomposition peaked at 700 °C, leading to severe strength loss [35,36].

Notably, specimens with higher NHT content demonstrated higher residual compressive strength at elevated temperatures. At 700 °C, the residual strength ratios for the Plain, NHTC-15, and NHTC-30 series were 23.6%, 22.4%, and 28.2%, respectively. This aligns with the study by Karatas et al. [19], which reported that while kaolin-incorporated mortar may have lower initial strength, it exhibits improved residual strength at high temperatures due to the increased pozzolanic reactivity of kaolin. Furthermore, Abdelmelek [22] noted that the optimal metakaolin replacement ratio for compressive strength depends on the W/B ratio, supporting the variations observed in this study.

The enhanced residual performance of the NHTC-30 series at 700 °C can be further explained by the phase transformation mechanism of clay minerals. According to Wang et al. [37], kaolinite (Al_2_O_3_∙2SiO_2_∙2H_2_O), the primary mineral in coal gangue and Hwangto, undergoes dehydroxylation within the temperature range of 550–750 °C. This process removes structural hydroxyl groups and collapses the crystalline lattice, transforming inert kaolinite into highly reactive amorphous metakaolin (Al_2_O_3_∙2SiO_2_). Although the NHT used in this study was non-calcined, the exposure to 700 °C during the fire test effectively acted as an “in situ thermal activation” process. The newly formed metakaolin then reacts with calcium hydroxide (Ca(OH)_2_) in the matrix to form secondary C-S-H and C-A-S-H gels, refining the pore structure and reinforcing the matrix. This mechanism aligns with the finding that thermally activated kaolinite-based materials significantly improve mechanical properties and microstructural density [37].

### 3.3. Stress–Strain Characteristics

**Figure 5:**
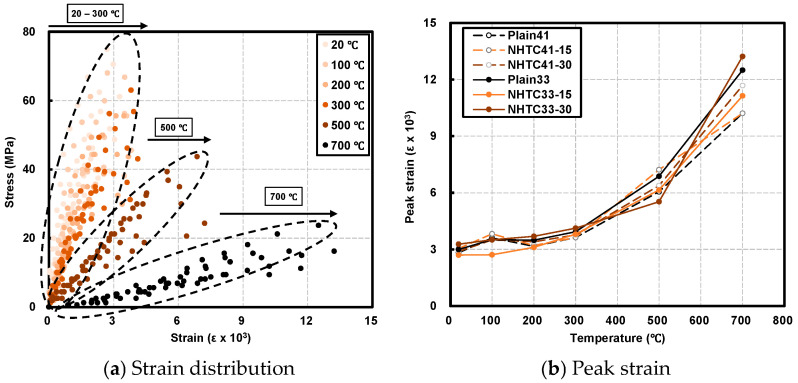
Overview of strain properties.

Figure 5a illustrates the stress–strain characteristics of the specimens, while Figure 5b presents the variations in peak strain. As the target temperature exceeded 300 °C, the slope of the stress–strain curve became noticeably flatter, indicating a reduction in stiffness due to decreased peak stress and increased strain.

In the temperature range of 20–300 °C, the peak strain remained relatively constant, ranging from 0.003 to 0.004. However, at temperatures above 500 °C, a significant increase was observed, reaching approximately 0.006–0.007 at 500 °C and 0.010–0.013 at 700 °C. Consequently, the specimens exhibited brittle failure characteristics below 300 °C, transitioning to a more ductile failure mode at temperatures above 500 °C [38,39].

**Figure 6:**
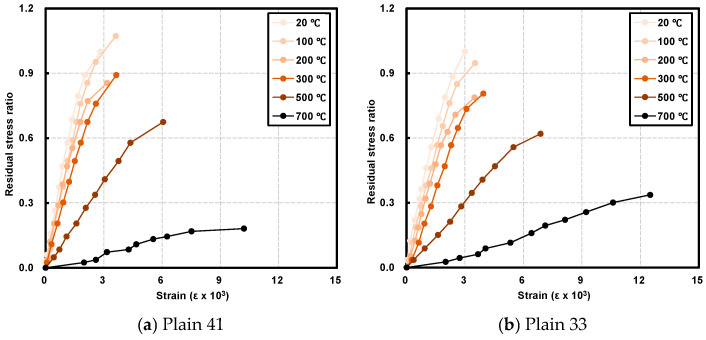

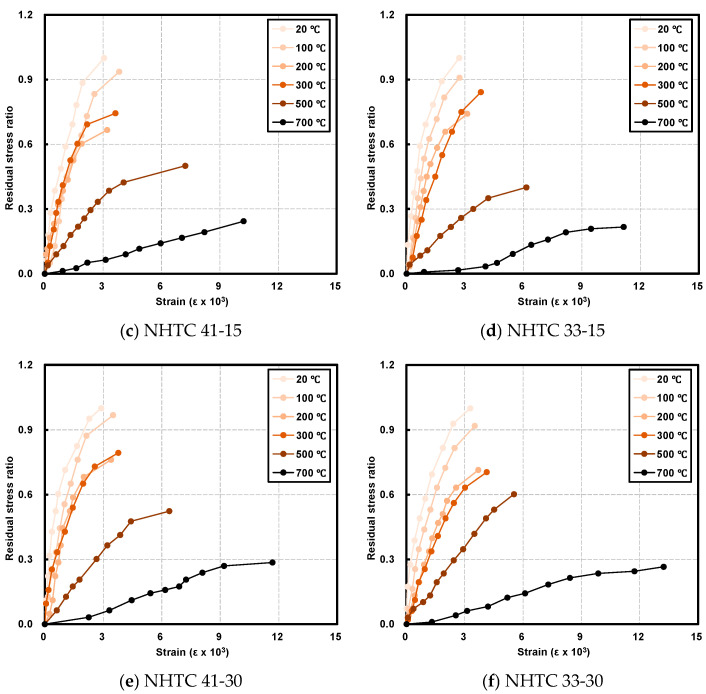
Stress–strain curve after exposure to target temperature.

Figure 6a–f show the stress–strain relationships of each specimen at the target temperatures. To interpret the deformation behavior, the damage evolution model proposed by Lin et al. [12] can be applied. They categorized the stress–strain curve into a “linear elasticity” stage, where strain increases linearly with loading, and an “elastoplasticity” stage, characterized by the loss of linearity due to micro-crack proliferation and macroscopic crack nucleation.

Applying this framework to the current study, distinct trends were observed with increasing temperature. As the target temperature rose, the linear elasticity region contracted, while the elastoplasticity region significantly expanded. Specifically, below 300 °C, linear behavior was maintained up to approximately 76.6–84.0% of the peak strain. However, at 500 °C and 700 °C, the linear limit retreated, and the quasi-linear (elastoplastic) behavior extended to ranges of approximately 52.3–88.9% and 60.4–90.0%, respectively. This prolongation of the elastoplastic phase indicates increased ductility driven by extensive crack development at elevated temperatures [38].

### 3.4. Dissipated Energy and Microstructure

**Figure 7:**
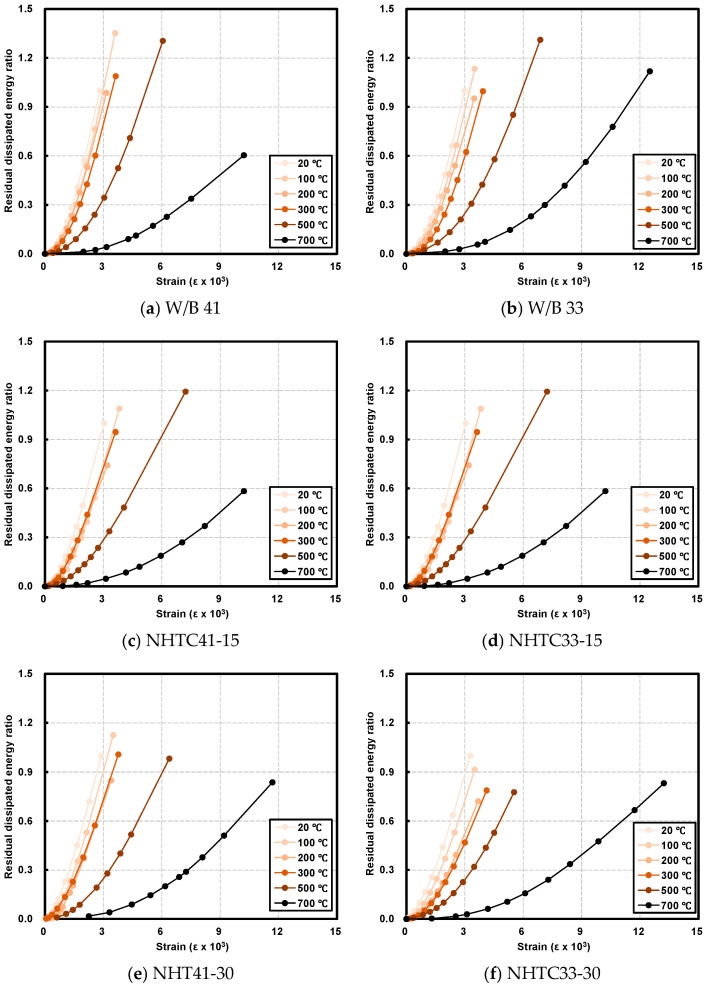
Dissipated energy after exposure to target temperature.

Table 5 summarizes the dissipated energy, while Figure 7 illustrates the relative dissipated energy trends at target temperatures. Generally, the relative dissipated energy decreased as the temperature increased. However, the NHT-incorporated series exhibited higher values compared to the Plain series, attributed to the increased ductility imparted by NHT.

In the W/B 41 series, the dissipated energy remained comparable to or higher than room temperature values up to 500 °C. Specifically, the Plain series recorded residual energy ratios of 134.7% (100 °C), 129.6% (500 °C), and 58.2% (700 °C). The NHTC-15 series showed 108.7–121.8% up to 500 °C, dropping to 57.6% at 700 °C. Notably, the NHTC-30 series maintained stable energy absorption, recording 112.7% at 100 °C and 83.8% at 700 °C. While the Plain specimen exhibited the highest energy up to 500 °C, the NHTC-30 specimen outperformed all others at 700 °C, demonstrating superior residual performance at extreme temperatures.

Conversely, in the W/B 33 series, the Plain specimen maintained high dissipated energy ratios (95.9–130.9%) across most temperatures and, unlike other specimens, showed an increase at 700 °C (112.2%). In contrast, the NHTC-15 and NHTC-30 series generally exhibited lower residual dissipated energy compared to room temperature. This discrepancy suggests that in lower-strength matrices (W/B 41), the relative contribution of NHT to energy retention is more pronounced than in high-strength matrices (W/B 33). However, at 700 °C, the NHTC-30 series consistently showed higher residual dissipated energy than the NHTC-15 series, confirming that higher NHT content contributes to enhanced energy absorption capacity under high-temperature conditions.

**Figure 8:**
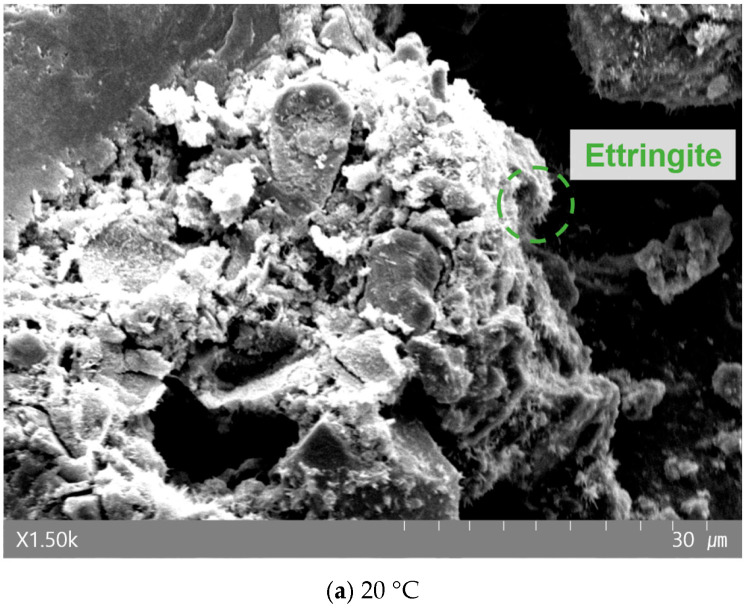

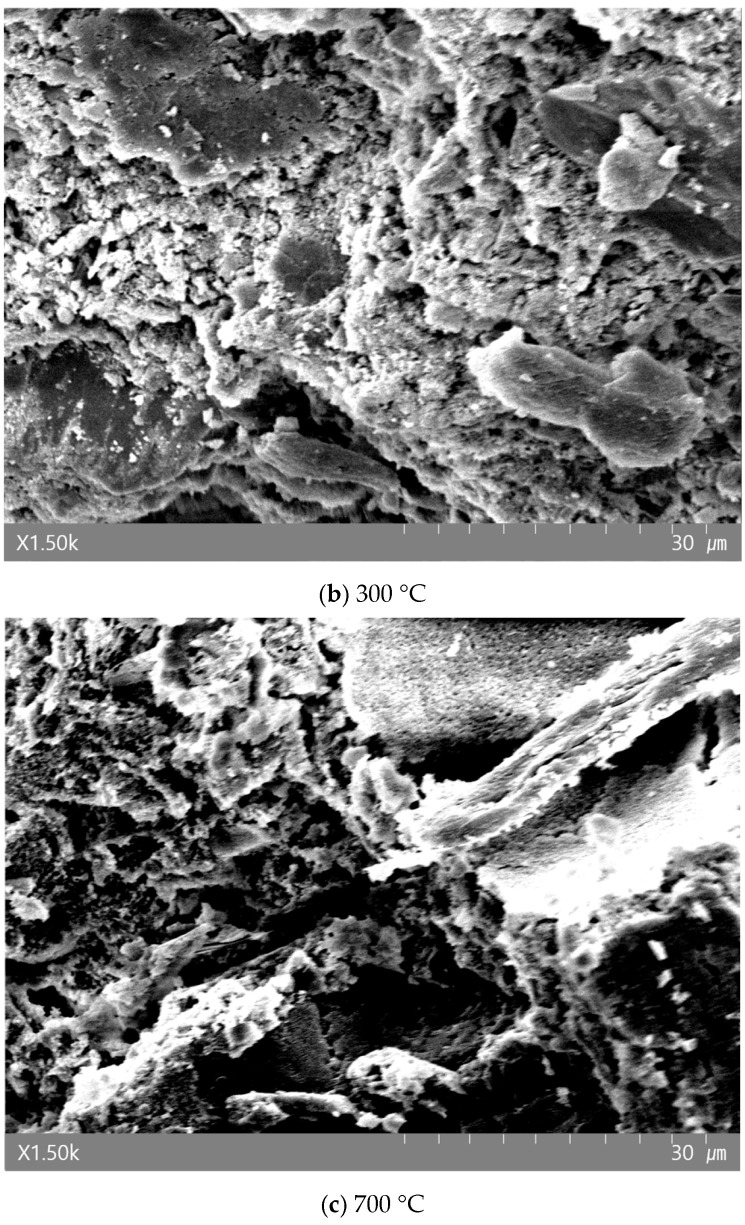
SEM image of Plain after exposure to each temperature.

**Figure 9:**
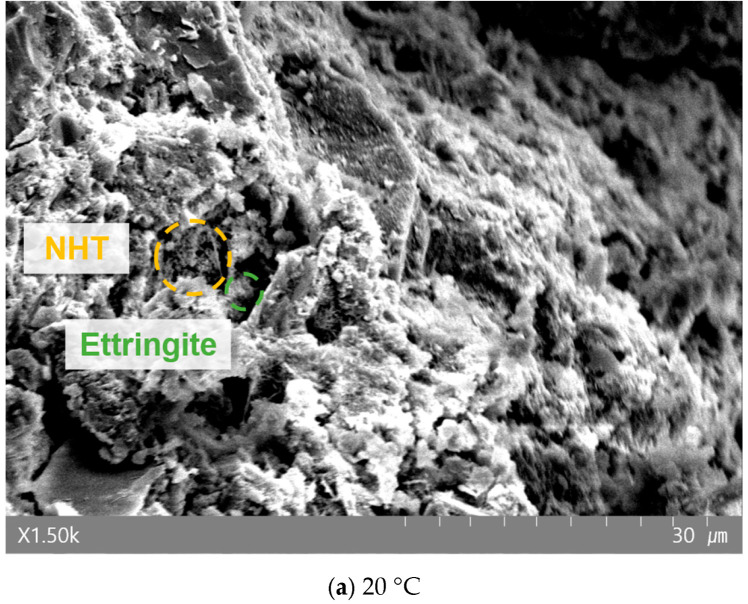

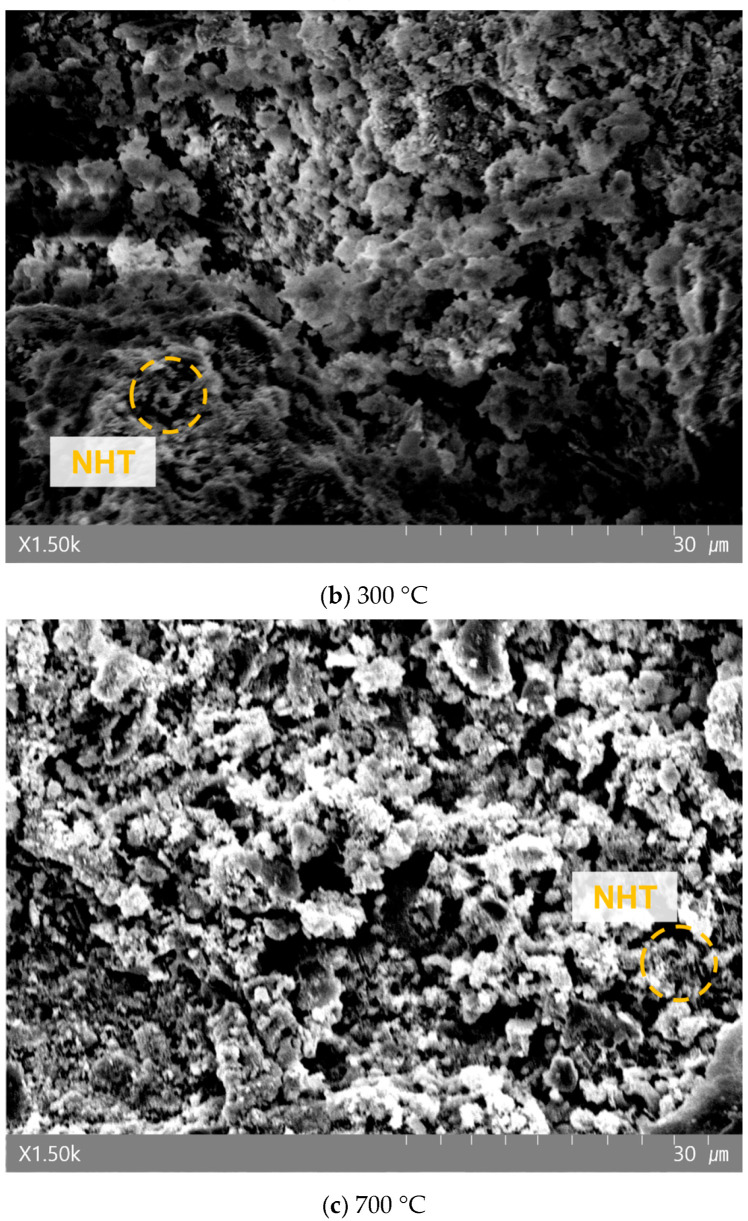
SEM image of NHTC-15 after exposure to each temperature.

**Figure 10:**
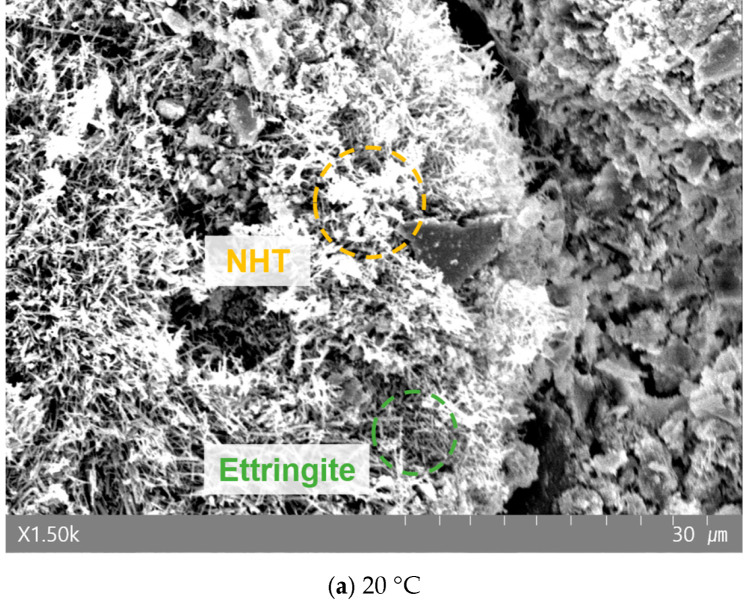

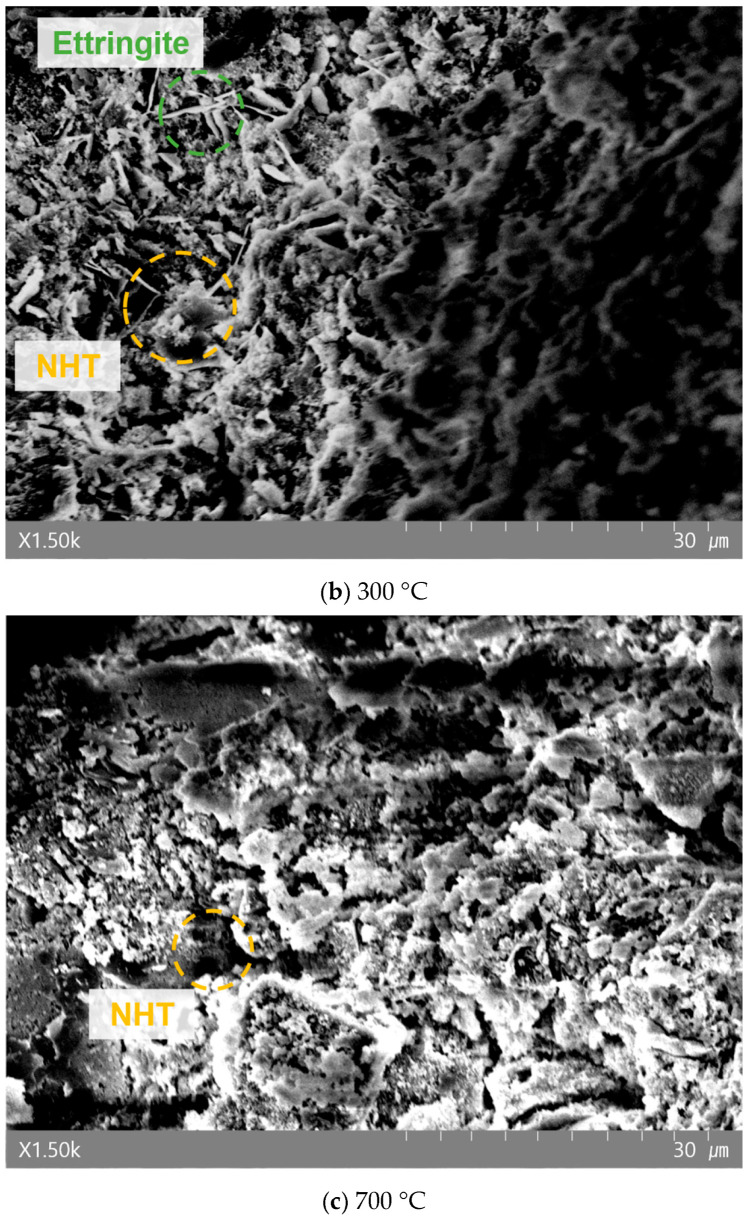
SEM image of NHTC-30 after exposure to each temperature.

Figure 8, Figure 9 and Figure 10 present the morphological changes in the microstructure analyzed via SEM. At 20 °C, needle-shaped crystals, identified as ettringite, were clearly observed with increasing NHT content. However, in specimens heated to 700 °C, these crystalline structures were absent. This is consistent with the established literature stating that ettringite thermally decomposes and loses its crystalline structure in the range of approximately 70–100 °C [40]. Typically, exposure to 700 °C induces significant coarsening of the pore structure and micro-cracking due to the dehydration of C–S–H gel. However, the SEM micrographs of the NHT-incorporated specimens at 700 °C exhibit a relatively more cohesive structure with fewer visible macro-cracks compared to the Plain specimen. This phenomenon can be attributed to the thermal transformation of kaolinite in NHT into metakaolin (approx. 450–600 °C), which may participate in secondary reactions or induce a localized sintering effect that partially fills the voids [15,41]. Although quantitative porosimetry (e.g., MIP) was not conducted in this study, these morphological observations provide qualitative support for the superior residual mechanical performance and energy dissipation capacity observed in the NHTC-30 series.

## 4. Conclusions

This study investigated the potential of using non-calcined Hwangto (NHT) as a sustainable mineral admixture to enhance the fire resistance of concrete. By evaluating the residual mechanical properties of concrete containing NHT under elevated temperatures up to 700 °C, this study provides critical insights into the “in situ activation” effect of clay-based materials during fire exposure. The key findings and contributions are summarized as follows:

1. Thermal Stability and Mass Retention: NHT incorporation effectively mitigated mass loss at elevated temperatures. Specifically, the NHTC-30 series exhibited the lowest unit weight reduction rate above 300 °C. This suggests that the inclusion of NHT contributes to maintaining the material’s physical stability under thermal stress, outperforming the Plain concrete in high-temperature ranges.
2. Mechanism of Residual Strength Enhancement: While non-calcined NHT resulted in lower initial strength at room temperature, it demonstrated a remarkable “strength reversal” capability at high temperatures. At 700 °C, the NHTC-30 series retained the highest residual compressive strength ratio (28.2%), surpassing the Plain series (23.6%). This improvement is attributed to the thermal transformation of kaolinite in NHT into reactive metakaolin at approximately 550–750 °C, which subsequently promoted pozzolanic reactions and densified the microstructure even under severe thermal degradation.
3. Ductility and Deformation Characteristics: The stress–strain analysis revealed a distinct transition from brittle to ductile failure modes with increasing temperature. The NHTC-30 series showed the highest peak strain at 700 °C, indicating superior deformability. This characteristic is crucial for preventing sudden structural collapse during fire incidents, thereby enhancing the overall safety margin of the structure.
4. Energy Dissipation Capacity: The energy absorption capability varied with the matrix strength (W/B ratio). Notably, in normal-strength concrete (W/B 41), the NHTC-30 series demonstrated the highest dissipated energy at 700 °C, confirming its superior post-peak resistance. This finding highlights that a sufficient replacement level of NHT (30%) is beneficial for maximizing energy absorption in fire scenarios.

In conclusion, this research demonstrates that non-calcined Hwangto is not merely an inert filler but a functional admixture that becomes thermally activated during fire exposure. By converting the weakness of “non-calcination” into a “high-temperature activation” advantage, NHT incorporation offers a sustainable solution for improving the residual structural integrity and fire safety of concrete structures.

## Figures and Tables

**Table 1:** Experimental program.

Classification	Program
Specimen dimension	Φ100 × 200 mm
Cement	Ordinary Portland Cement
Mineral admixture	Non-calcined Hwangto (0%, 15%, 30%)
W/B	41.3, 33.0
Curing conditions	Water; Room temperature: 20 ± 2 °C; Humidity: 60 ± 5%
Temperature	20, 100, 200, 300, 500, 700 °C
Heating rate	1 °C/min
Holding time	60 min
Test items	Unit weight, Compressive strength, Stress–strain, Dissipated energy

**Table 2:** The physical properties of the materials [27].

Materials	Properties
Cement	Type I Ordinary Portland Cement Density: 3.15 g/cm^3^; Fineness: 3200 cm^2^/g
Mineral admixture	Non-calcined Hwangto Density: 2.50 g/cm^3^; Fineness: 3300 cm^2^/g
Coarse aggregate	Crushed granite aggregate Density: 2.68 g/cm^3^; Fineness modulus: 7.03 Absorption: 0.68%; Maximum size: 20 mm
Fine aggregate	River sand Density: 2.54 g/cm^3^; Fineness modulus: 2.54 Absorption: 1.6%
Superplasticizer	Polycarboxylic-based acid

**Table 3:** Chemical properties of binder.

Materials	Chemical Composition (%)								
	CaO	SiO_2_	Al_2_O_3_	Fe_2_O_3_	MgO	SO_3_	K_2_O	Others	L.O.I ^1^
OPC	60.34	19.82	4.85	3.30	3.83	2.88	1.08	0.86	3.02
NHT ^2^	0.93	40.00	32.90	7.79	1.54	-	0.76	16.62	13.7

^1^ Loss of ignition. ^2^ Non-calcined Hwangto.

**Table 4:** Mix proportions of Plain and NHTC.

ID	W/B	S/a	Unit Weight (kg/m^3^)				
			W	C	NHT	S	G
Plain41	41.3	46.0	165	400	-	799	758
NHTC41-15				340	60	794	752
NHTC41-30				280	120	788	747
Plain33	33.0	43.0		500	-	711	762
NHTC33-15				425	75	705	755
NHTC33-30				350	150	699	748

**Table 5:** Dissipated energy after exposure to target temperature.

Temperature (°C)	Dissipated Energy (KPa)					
	Plain41	NHTC41-15	NHTC41-30	Plain33	NHTC33-15	NHTC33-30
20	93.0 ± 5.8 ^(1)^ [63.6] ^(2)^	101.8 ± 3.0 [68.7]	83.3 ± 0.8 [73.2]	126.3 ± 2.0 [59.9]	144.5 ± 2.3 [71.4]	143.4 ± 1.7 [71.5]
100	125.3 ± 1.6 [62.4]	110.7 ± 4.0 [63.6]	93.8 ± 0.3 [70.4]	144.3 ± 0.6 [61.5]	122.4 ± 1.9 [66.3]	130.9 ± 1.8 [66.4]
200	91.2 ± 4.6 [65.0]	75.2 ± 0.4 [72.3]	71.4 ± 1.7 [70.2]	121.1 ± 3.6 [62.6]	117.5 ± 5.9 [68.0]	130.5 ± 5.2 [64.1]
300	100.0 ± 1.7 [59.5]	95.4 ± 2.3 [72.6]	83.9 ± 0.5 [71.4]	125.9 ± 1.6 [56.3]	148.7 ± 4.0 [61.9]	113.2 ± 4.5 [63.8]
500	120.5 ± 9.2 [57.0]	124.0 ± 2.1 [70.6]	81.6 ± 3.3 [62.0]	165.3 ± 8.3 [55.0]	118.3 ± 5.9 [64.3]	112.2 ± 5.6 [55.1]
700	54.1 ± 1.6 [56.6]	58.6 ± 2.0 [48.4]	69.8 ± 1.8 [53.1]	141.7 ± 3.4 [47.8]	86.4 ± 4.3 [47.8]	119.3 ± 6.0 [55.6]

^(1)^ Dissipated energy (KPa); ^(2)^ relative dissipated energy ratio (%).

## Data Availability

The original contributions presented in this study are included in the article. Further inquiries can be directed to the corresponding author.
